# Physician Documentation of Sepsis Syndrome Is Associated with More Aggressive Treatment

**DOI:** 10.5811/westjem.2015.3.25529

**Published:** 2015-04-10

**Authors:** Lisa R. Stoneking, John P. Winkler, Lawrence A. DeLuca, Uwe Stolz, Aaron Stutz, Jenifer C. Luman, Michael Gaub, Donna M. Wolk, Albert B. Fiorello, Kurt R. Denninghoff

**Affiliations:** * The University of Arizona College of Medicine, Department of Emergency Medicine, Tucson, Arizona; † Swedish Medical Center, Department of Emergency Medicine, Denver, Colorado; ‡ Fairchild Medical Center, Yreka, California; § EMA, Alvarado Hospital Emergency Department, San Diego, California; ¶ Scripps Encinitas Emergency Department, Encinitas, California; || Geisinger Health System, Department of Laboratory Medicine, Danville, Pennsylvania

## Abstract

**Introduction:**

Timely recognition and treatment of sepsis improves survival. The objective is to examine the association between recognition of sepsis and timeliness of treatments.

**Methods:**

We identified a retrospective cohort of emergency department (ED) patients with positive blood cultures from May 2007 to January 2009, and reviewed vital signs, imaging, laboratory data, and physician/nursing charts. Patients who met systemic inflammatory response syndrome (SIRS) criteria and had evidence of infection available to the treating clinician at the time of the encounter were classified as having sepsis. Patients were dichotomized as RECOGNIZED if sepsis was explicitly articulated in the patient record or if a sepsis order set was launched, or as UNRECOGNIZED if neither of these two criteria were met. We used median regression to compare time to antibiotic administration and total volume of fluid resuscitation between groups, controlling for age, sex, and sepsis severity.

**Results:**

SIRS criteria were present in 228/315 (72.4%) cases. Our record review identified sepsis syndromes in 214 (67.9%) cases of which 118 (55.1%) had sepsis, 64 (29.9%) had severe sepsis, and 32 (15.0%) had septic shock. The treating team contemplated sepsis (RECOGNIZED) in 123 (57.6%) patients. Compared to the UNRECOGNIZED group, the RECOGNIZED group had a higher use of antibiotics in the ED (91.9 vs.75.8%, p=0.002), more patients aged 60 years or older (56.9 vs. 33.0%, p=0.001), and more severe cases (septic shock: 18.7 vs. 9.9%, severe sepsis: 39.0 vs.17.6%, sepsis: 42.3 vs.72.5%; p<0.001). The median time to antibiotic (minutes) was lower in the RECOGNIZED (142) versus UNRECOGNIZED (229) group, with an adjusted median difference of −74 minutes (95% CI [−128 to −19]). The median total volume of fluid resuscitation (mL) was higher in the RECOGNIZED (1,600 mL) compared to the UNRECOGNIZED (1,000 mL) group. However, the adjusted median difference was not statistically significant: 262 mL (95% CI [ −171 to 694 mL]).

**Conclusion:**

Patients whose emergency physicians articulated sepsis syndrome in their documentation or who launched the sepsis order set received antibiotics sooner and received more total volume of fluid. Age <60 and absence of fever are factors associated with lack of recognition of sepsis cases.

## INTRODUCTION

The early identification of sepsis leads to timely initiation of antibiotics and fluid resuscitation.^1^,^2^ Administration of empiric, broad-spectrum antibiotic therapy is recommended within the first hour of recognition of severe sepsis or septic shock because it has been shown to decrease sepsis-related mortality.^3^ Indeed, each hour of delay in the administration of antibiotic therapy is associated with an increased mortality rate.^4^,^5^ However, despite the focus on improving care for sepsis patients, key questions remain unanswered. Does consideration of the sepsis syndrome – as distinct from localized infection or other diagnoses such as dehydration - have an independent effect on subsequent interventions and therapies delivered? After working diligently on a sepsis-screening tool, Moore stated, “early recognition of sepsis was a major obstacle to protocol implementation…. [and we hypothesize that] aggressive screening for sepsis would improve early recognition…and decrease sepsis-related mortality.”^6^ This may be particularly relevant in emergency department (ED) patients who present with a relatively complicated clinical picture, have impediments to diagnosis, such as altered mental status, have sepsis without fever, have undifferentiated shock or compensated shock.

In this study, we tested the hypothesis that consideration and documentation of sepsis syndromes by the emergency physician reduces the time to antibiotic administration and affects the amount of fluid resuscitation delivered to patients with sepsis.

## METHODS

We conducted the study using patient data from a large, urban academic ED with an annual volume of approximately 70,000 patients. Greater than 4,000 blood cultures are ordered annually from the ED with an 8.5% positive rate. We identified a retrospective cohort of patients who presented to the ED and had bacteria cultivated from blood cultures (i.e., blood cultures were “positive”) that were drawn during their ED visit over a 20-month period using a pathology database.

We used patient vital signs and initial laboratory studies to identify which of these patients met systemic inflammatory response syndrome (SIRS) criteria.^7^ Patients who met SIRS criteria and had clinical, laboratory, or radiographic evidence of infection available to the treating clinician at the time of the encounter were classified as having sepsis. We classified patients who were septic and who experienced an episode of hypotension, or other signs of organ dysfunction as having severe sepsis.^8^ Patients who were hypotensive on initial presentation and remained hypotensive after an initial fluid bolus were classified as having septic shock. We used the 2004 Sepsis guidelines in this classification system given that some of the patients included in the analysis were pre-2008.^3^,^8^

**Figure 1:**
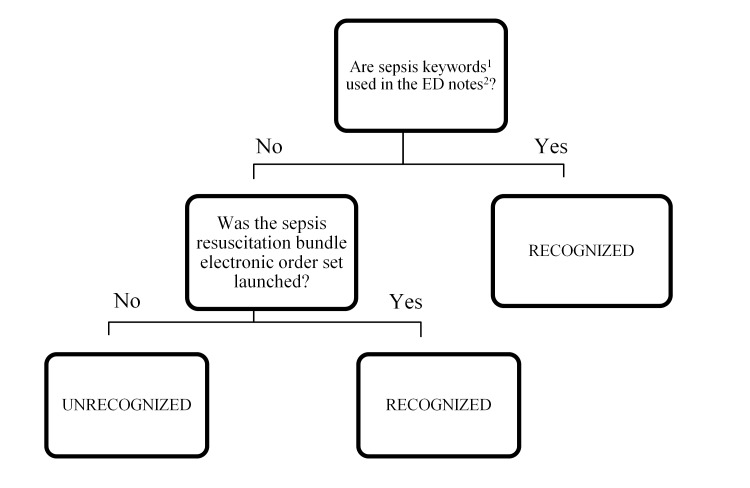
Algorithm for retrospective identification of sepsis recognition by emergency physicians. *ED*, emergency department ^1^Sepsis keywords include systemic inflammatory response syndrome (SIRS), sepsis, severe sepsis, septic shock, septicemia, and septic. ^2^ED notes include attending’s or resident’s differential diagnosis, medical decision-making, ED course notes, or clinical impression.

We reviewed handwritten and electronic physician and nursing charts for consideration of a sepsis syndrome. Patient sepsis was considered RECOGNIZED if there was documentation of consideration of sepsis, severe sepsis, or septic shock in the attending or resident differential diagnosis, in the documentation of the ED course, in the final diagnosis, or by initiation of the sepsis resuscitation bundle electronic order set. The remaining patients were classified as UNRECOGNIZED (Figure 1). Because lactate is an independent predictor of mortality in infected and non-infected admitted elderly patients^9^ and is often ordered for patients where the treating clinician doesn’t suspect sepsis, it was not used to determine if a patient was considered as RECOGNIZED or UNRECOGNIZED.

To assess the inter-rater agreement for categorizing a patient as either RECOGNIZED or UNRECOGNIZED from medical record reviews, a second group of evaluators reviewed a randomly selected subset of 103 of the charts. The kappa statistic was used to assess inter-rater agreement and agreement was considered adequate if the lower limit of the 95% confidence interval was above 0.61, the threshold for “substantial” agreement.^10^

Summary statistics for continuous data are presented as medians and IQRs and proportions are presented as percentages with 95% CIs using the Pearson-Clopper “exact” method. We used Fisher’s exact test to compare proportions and median regression to compare continuous variables. *A priori*, α was set at ≤ 0.05. To test our hypotheses, we used multivariable median regression to calculate medians and absolute median differences, along with 95% CIs, for volume of fluid administration and time to antibiotic between RECOGNIZED and UNRECOGNIZED groups. We controlled for patient age, sex, and sepsis severity. Covariates were included in the final model if either they were significantly associated with the outcome variable (p≤0.05) or if they were judged a significant confounder of the relationship between being RECOGNIZED/UNRECOGNIZED and the outcome variable. We considered covariates significant confounders if their inclusion changed the regression coefficient (median difference) for the RECOGNIZED/UNRECOGNIZED variable by greater than 10%. Median differences were considered statistically significant if the 95% CI did not contain 0. We performed all analyses using Stata (v.12.1, Stata Corp., College Station, Texas). Approval for the study was obtained from University of Arizona Institutional Review Board.

## RESULTS

Table 1 shows population characteristics and demographics. A total of 315 positive blood cultures were identified between May 2007 and January 2009. SIRS criteria were present in 228/315 cases (72.4%, 95% CI [67.1 – 77.2]). Our chart review identified sepsis syndromes in 214/315 cases (67.9%, 95% CI [62.5 – 73.1]). Of the 214 septic patients, 118 (37.5%, 95% CI [32.1 – 43.1]) had sepsis, 64 (20.3%, 95% CI [16.0 – 25.2]) had severe sepsis, and 32 (10.2%, 95% CI [7.1 – 14.0]) had septic shock. The treating team recognized sepsis in 123/214 (57.5%, 95% CI [50.6 – 64.2]) patients.

Table 2 shows the comparison of characteristics of the RECOGNIZED vs. UNRECOGNIZED group. Antibiotic use, age, distribution of sepsis, and fluid administration differed significantly between the RECOGNIZED and UNRECOGNIZED group. Patients in the RECOGNIZED group tended to be older, had greater sepsis severity, more fluid administered, higher proportion of antibiotic administration, and shorter time to antibiotic administration than those in the UNRECOGNIZED group.

**Figure 2:**
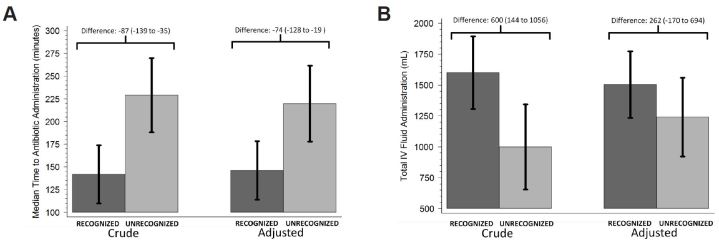
*A*, Comparison of crude and adjusted medians and differences between RECOGNIZED (dark gray) and UNRECOGNIZED (light gray) groups for time to antibiotic administration and *B*, total intravenous (IV) fluid administration in the emergency department. Bars indicate 95% CIs for medians. Median differences (95% CIs) are reported above each comparison. We calculated medians and median differences, along with 95% CIs, using median regression. Adjusted values were calculated using multivariable analyses adjusting for patient age, sex, and sepsis severity (septic, severe sepsis, or septic shock).

Figure 2 shows both the crude and adjusted medians and differences, along with 95% CIs, comparing the RECOGNIZED and UNRECOGNIZED groups for time to antibiotic administration in the ED and total volume of intravenous fluid administered. The median time (minutes) to antibiotic administration from triage time was significantly lower in the RECOGNIZED versus the UNRECOGNIZED group (142 versus 229; crude difference: −87 (95% CI [−139 – −35]). In the adjusted analysis (controlling for patient age, sex, and sepsis severity), time to antibiotic administration remained significantly lower in the RECOGNIZED group (median difference = −74 minutes, 95% CI [−128 – −19]). Patient sex (p=0.57), age (p=0.30), and sepsis severity (p=0.3) were not significantly associated with time to antibiotic but were significant confounders for the relationship between RECOGNIZED/UNRECOGNIZED and time to antibiotic administration.

The total median volume of fluid resuscitation (mL) was significantly greater in the RECOGNIZED compared to the UNRECOGNIZED group (1600 vs. 1000; crude median difference: 600, 95% CI [283 – 1,197]; Figure 2). However, after controlling for patient age, sex, and sepsis severity, total fluid administration did not differ statistically between the two groups (median difference: −262 mL, 95% CI [−171 – 694]). Sex (p=0.81) and age (p=0.073) were not significantly related to total fluid volume but were significant confounders. Sepsis severity, however, was significantly related to total fluid administered (p<0.001), with patients with severe sepsis getting a median of 1,632 mL (95% CI [1037 – 2,227]) of additional fluid compared to those with sepsis.

Overall agreement was 89.3% for independent reviewers classifying patients as RECOGNIZED vs. UNRECOGNIZED and the inter-rater reliability (kappa statistic) was 0.78 (95% CI [0.66 – 0.90]), indicating substantial agreement.^10^

## DISCUSSION

Even with aggressive therapy, sepsis is a condition that is associated with high mortality. Without the consideration of sepsis syndrome in the differential diagnosis, or with late consideration of this disease process, antibiotic administration and fluid resuscitation may be delayed. Without fever at triage presentation, this syndrome is even easier to overlook. In our study, septic patients in the RECOGNIZED group had a fever 47.2% of the time compared to only 30.8% of the time in the UNRECOGNIZED group (p=0.017).

To improve survival from sepsis, it must be promptly recognized and then expeditiously and aggressively treated.^1^ The difference between Rivers’ original goal-directed intervention and control groups was not in the types of treatments administered, but merely in the speed with which each group using the same tools achieved therapeutic endpoints.^1^ Multiple other studies demonstrate improved outcomes with identification and aggressive treatment of septic patients.^11^–^14^

There were several reasons for selection of the ED cohort of patients with positive blood cultures. First, bacteremia offered a consistent and reliable means of identifying patients who were truly infected with a bacterial illness that had a high likely progression to severe sepsis and septic shock. Second, antibiotic time and fluid administration volume totals are reliably recorded in nursing documentation in the ED.

We found that over 42% of patients who met sepsis criteria in our retrospective analysis did not have sepsis syndromes explicitly articulated as part of the ED record by the treating physicians, nor did they have the sepsis bundle initiated. We also found that for the 58% of patients where sepsis was overtly considered by the treating team, there was a significant decrease in time to delivery of antibiotic therapy. This difference persisted even after controlling for age, sex, and severity of sepsis. Time to antibiotic administration has been repeatedly demonstrated to have a significant impact on mortality.^4^ This effect is even more pronounced for patients with severe sepsis than those with septic shock.^5^ Patients with severe sepsis are generally less overtly ill than patients with septic shock when they present to the ED; therefore, the urgency of treatment for this group may be underappreciated. Although the median total intravenous fluid administration did not differ significantly between the two groups in the adjusted analysis, there was still a trend toward higher volumes in the RECOGNIZED group. This may have been a reaction by the treating team to worsening symptoms over time. However, time to antibiotic treatment, while lower in the RECOGNIZED group, was not significantly related to sepsis severity and thus may have reflected proactive treatment by the treating team after consideration of sepsis.

We demonstrate that sepsis syndromes were explicitly identified in our cohort only 58% of the time. While a retrospective design precludes our ability to determine whether this is solely a documentation issue, an explicit failure to recognize sepsis as the cause of the patient’s illness, or a combination of the two, it is clear that for those individuals who were identified as potentially septic, the course of their treatment was altered by that diagnostic impression. There were only eight cases identified in which the sole evidence of recognition of sepsis syndrome was launching the sepsis resuscitation bundle. However, given the labor-intensive nature of the bundle, we conclude that the clinician “recognized” sepsis prior to bundle initiation.

The UNRECOGNIZED group had less sick patients (i.e. a lower proportion of severe sepsis or septic shock compared to the RECOGNIZED group) and tended to be younger. This may contribute to the lower rates of recognition in this group. Patients with shock and those who fall on the sicker end of the illness spectrum are almost certainly easier to recognize. However, our study population was composed entirely of bacteremic patients who were classified as having sepsis by objective, well-recognized clinical parameters. In other words, patients categorized as UNRECOGNIZED were still very sick and required prompt treatment for sepsis. All septic patients require early antibiotic administration and many also frequently require fluid resuscitation to prevent progression to severe sepsis and shock. It is therefore important to recognize patients along the entire clinical spectrum from early sepsis to septic shock in order to optimize their care in the ED and maximize their chances for survival. However, even after controlling for sepsis severity, those in the RECOGNIZED group still had a shorter time to antibiotic administration. This suggested to the authors that it was the consideration by the treating physicians that resulted in shorter time to antibiotic administration and not simply because this group tended to be sicker on average.

Additionally complicating the clinical picture is the fact that blood cultures may be slow to yield a causative organism, and may have limited sensitivity for organisms that do not grow well in blood culture media.^15^ In fact, up to 20–50% of bloodstream infections may not be identified by routine blood culture methods.^16^ Identification of false positive blood cultures more often relies on the epidemiologic data obtained from blood cultures from a given laboratory rather than the clinical context of a given patient. For example, while *S. epidermidis* may be a common skin contaminant, it may also be the result of a skin infection with hematologic spread. The three most commonly identified organisms in this pathology database were *S. aureus, E. coli, and S. viridans.*

Difficulty with the identification of sepsis persists in our clinical environment despite a Surviving Sepsis Campaign (SSC) committee at our hospital, a SSC bundle electronic order set, and multiple emergency physician “champions” of sepsis. In the era of electronic medical records, perhaps a computer-integrated sepsis ID tool could help identify those patients previously UNRECOGNIZED and prompt physicians to consider the diagnosis.

Recent trials such as ProCESS^2^ and ARISE^17^ have invigorated the discussion about optimal sepsis care. However, the difference of opinion between Rivers and subsequent investigators has not been over the importance of prompt antibiotic administration and fluid resuscitation, but rather on the method used to determine resuscitative endpoints.

## LIMITATIONS

We recognize that there are several important limitations to this study. It is a single-center study performed in an urban academic setting and not powered to detect significant differences in survival between the RECOGNIZED and UNRECOGNIZED groups. However, the only death in the ED was in the UNRECOGNIZED group. Because of the setting it may not be generalizable to suburban or rural venues.

This was a retrospective chart review and follow-up telephone calls to assess survival beyond hospital discharge were not feasible. Survival to discharge was not our main outcome, as we knew we would not have enough power for analysis of survival.

There are well-known limitations to the process of extracting data from handwritten charts. There can be conflicting data in documentation between which interventions are ordered and which appear to have been completed by the nursing staff. Additionally, sepsis may have been considered by the treating team, but not documented by name and the SSC bundle may not have been initiated because of contraindications for individual patients. There is also the possibility of bias in our sample selection. Both false positive and false negative cultures are potential confounders in this study. Only patients who had blood cultures obtained that subsequently were positive were captured, thereby excluding septic patients with false negative blood cultures. There may have been unrecognized septic patients who did not have cultures obtained or whose cultures were negative, who were not included in this study.

Illness severity scores (Acute Physiology and Chronic Health Evaluation, Sequential Organ Failure Assessment, Simplified Acute Physiology Score) were not calculated, as much of this information is not available to the ED physician. Calculations of these scores require data based on the first 24 hours of hospital admission. This makes it difficult to determine the meaning of such scores during the initial ED encounter.

Additionally, this study used a pathology database of positive blood cultures from eight years ago that is no longer maintained. There have been great advances and education in sepsis care over the last decade, which may also limit this study. Hopefully clinicians today are better equipped to identify patients with sepsis syndrome earlier in their treatment course.

## CONCLUSION

Lack of documentation of sepsis in the physician chart was associated with increased time to antibiotic delivery and a smaller total volume of fluid administration in patients that were bacteremic and had clinical signs of sepsis syndrome. Increasing early recognition and documentation of sepsis may improve clinical outcomes by shortening the time to antibiotic treatment and increasing fluid administration. Age <60 and absence of fever are factors associated with lack of recognition of sepsis cases.

## Figures and Tables

**Table 1:** Demographics and characteristics of study population of patients who had bacteria cultivated from blood cultures.

Characteristic	N	Percent (95% CI)
Total	315	100
Age – years, median (IQR)	315	55 (38 – 71)
Patients 60+ years old	139	44.1 (38.6 – 49.8)
Male sex	181	57.5 (51.8 – 63.0)
ED disposition	315	
Admitted	242	76.8 (71.8 – 81.4)
Discharged	39	12.4 (9.0 – 16.5)<
Transferred	30	9.5 (6.5 – 13.3)<
Left AMA	3	1.0 (0.2 – 2.8)<
Died	1	0.3 (0.01 – 1.8)
Met SIRS criteria	228	72.4 (67.1 – 77.2)
Met sepsis criteria	214	67.9 (62.5 – 73.1)
Presence of fever (>38°C)	101	32.1 (26.9 – 37.5)
Sepsis severity	214	100
Sepsis	118	55.1 (48.2 – 61.9)
Severe sepsis	64	29.9 (23.9 – 36.5)
Septic shock	32	15.0 (10.5 – 20.4)
Sepsis recognized (RECOGNIZED)	123/214	57.6 (50.7 – 64.2)
Received antibiotic in ED – all patients	227	70.5 (65.2 – 75.4)
Time – antibiotic – minutes, median (IQR)	227	176 (107 – 320)
Received antibiotic in ED – septic patients	182/214	85.1 (79.6 – 89.5)
Time – antibiotic – minutes, median (IQR)	182	160 (100 – 310)
Received IV fluid – all patients	259	82.2 (77.5 – 86.3)
Volume of IV Fluid – mL, median (IQR)	253	1,000 (250 – 2000)
Received IV fluid – Septic patients	193/214	90.2 (85.4 – 93.4)
Volume of IV fluid – mL, median (IQR)	188	1,050 (500 – 2000)

*ED*, emergency department; *AMA*, against medical advice; SIRS, systemic inflammatory response system; *IV*, intravenous

**Table 2:** Characteristics of patients with sepsis by RECOGNIZED vs. UNRECOGNIZED status.

Characteristics	RECOGNIZED n=123	UNRECOGNIZED n=91	p-value
Age – years, median (IQR)	63 (58–68)	51 (45–57)	0.003
Patients 60+ years old, No. (%)	70 (56.9)	30 (33.0)	0.001
Male sex, No. (%)	66 (53.7)	56 (61.5)	0.27
ED disposition, No. (%)			0.33
Admitted	108 (87.8)	73 (80.2)	
Discharged	5 (4.1)	6 (6.6)	
Transferred	10(8.1)	11 (12.1)	
Died	0 (0)	1 (1.1)	
Presence of fever, No. (%)	58 (47.2)	28 (30.8)	0.017
Sepsis severity, No. (%)			<0.001
Sepsis	52 (42.3)	52 (72.5)	
Severe sepsis	48 (39.0)	48 (17.6)	
Septic shock	23 (18.7)	23 (9.9)	
Received sntibiotic in ED, No. (%)	113 (91.9)	69 (75.8)	0.002
Time to antibiotic – minutes, median (IQR)	142 (90–260)	229 (130–352)	0.002
Received IV Fluid, No. (%)	121 (98.4)	88 (96.7)	0.65
Volume of IV Fluid – mL, median (IQR)	1,600 (920–3000)	1,000 (355–2000)	<0.001

*ED*, emergency department; *IV*, intravenous
